# Dimensions of Human-Machine Combination: Prompting the Development of Deployable Intelligent Decision Systems for Situated Clinical Contexts

**DOI:** 10.1007/s10606-025-09514-4

**Published:** 2025-04-14

**Authors:** Ben Wilson, Chiara Natali, Matt Roach, Darren Scott, Alma Rahat, David Rawlinson, Federico Cabitza

**Affiliations:** 1 Swansea University, Computer Science, Swansea, Wales SA1 8EN UK; 2 Dipartimento di Informatica, Sistemistica e Comunicazione, Università degli Studi di Milano-Bicocca, Viale Sarca, 336, 20126 Milano, Italy; 3 School of Computer Science and Informatics, Cardiff University, Cardiff, Wales CF24 4AG UK; 4 NHS, EMRTS Cymru, Llanelli, Wales SA14 UK; 5 IRCCS Ospedale Galeazzi-Sant’Ambrogio, Milan, Italy

**Keywords:** Human-AI interaction, Human-centered AI, Hybrid intelligence, Real-world evaluation, Medical AI

## Abstract

Whilst it is commonly reported that healthcare is set to benefit from advances in Artificial Intelligence (AI), there is a consensus that, for clinical AI, a gulf exists between conception and implementation. Here we advocate the increased use of situated design and evaluation to close this gap, showing that in the literature there are comparatively few prospective situated studies. Focusing on the combined human-machine decision-making process - modelling, exchanging and resolving - we highlight the need for advances in exchanging and resolving. We present a novel relational space - *contextual dimensions of combination* - a means by which researchers, developers and clinicians can begin to frame the issues that must be addressed in order to close the chasm. We introduce a space of eight initial dimensions, namely *participating agents*, *control relations*, *task overlap*, *temporal patterning*, *informational proximity*, *informational overlap*, *input influence* and *output representation coverage*. We propose that our awareness of where we are in this space of combination will drive the development of interactions and the designs of AI models themselves. Designs that take account of how user-centered they will need to be for their performance to be translated into societal and individual benefit.

## Introduction

Informed clinical commentators say that healthcare looks set to benefit from widely reported advances in Artificial Intelligence (AI) (Keane and Topol 2018; Topol 2019). This expectation has been the subject of huge anticipation, review and investment from governments and societies across the globe (OECD 2021; Veale et al. 2023). However, clinical journals comment that inflated expectations surrounding machine learning have been around for some time, and the pace of translation into practice has lagged far behind (Chen and Asch 2017). In fact, as far back as 2016 doctors noted that the promise of big data to transform medicine was already considered old news (Obermeyer and Emanuel 2016). In 2019, *The Lancet* continued to report concerns of how AI in clinical medicine was overhyped (Collins et al. 2019). At the time, Google Health specialists admitted that there were limited examples of AI applications being successfully deployed (Kelly et al. 2019).

In general, there has been a consensus that, for clinical AI, there is a gulf between conception and implementation (Keane and Topol 2018). A *Nature Medicine* paper noted that very few algorithms had reached clinical deployment in a way that was ‘challenging the balance between hope and hype’ (Laak et al. 2021, p775). In an editorial in *Nature Digital Medicine*, Keane and Topol bemoaned the lack of awareness of clinicians about the *AI chasm* which they said existed between algorithm development and meaningful real-world applications (Keane and Topol 2018).

Most of the commentary on the *AI chasm* focuses on the mismatch between the prevailing approaches used for the technical evaluation of Machine Learning (ML) & AI and those used to evaluate their impact in clinical settings. A comment piece in *Nature Digital Medicine* argued that what is needed is “a concerted effort around not just the creation, but also the delivery of AI" (Li et al. 2020, p1). A review in 2021 in the *New England Journal of Medicine* warned that, “few studies have prospectively evaluated the implementation of machine learning (e.g., using a clinical endpoint instead of a statistical endpoint)" (Ganguli et al. 2020, p3). The need for better translation of AI performance to clinical impact was heavily underlined when *Nature Machine Intelligence* published a review of 2.2k papers that had directed the power of ML against covid-19 the previous year. The review concluded that none of the identified models had potential clinical use due to methodological flaws and/or underlying biases (Roberts et al. 2021).

Despite recent trends showing an increase in research into Human-AI collaboration and human-machine hybrid approaches, there is little that deeply blends the strengths of humans and computers in combined decision-making. In this paper, we attempt to get beneath the mismatch between expectation and delivery and uncover factors that could contribute to this implementation problem. We identify two issues.

First, as noted by those lamenting the *AI chasm*, the dominant method of evaluation lacks focus on *clinical* performance.

Second, and related, there is missing from the computer science community, an approach to architecting and developing AI models that anticipate and take account of how user-centered they will need to be in deployment for their performance to be translated into real benefit in combined decision-making.

We argue that evaluation needs to be formative and situated in realistic workflows. Furthermore, appropriate development will require the design of a more sophisticated peer space, where humans and algorithms work more closely in decision-making processes.

For this to happen, there must be greater awareness of the commonalities and differences in the resources and approaches that humans and algorithms bring to the task - in other words, their modes of combination.

There must also be opportunities for greater dialogue between them: this inevitably requires the yet further development of machines, enabling their access to mutually tractable representations.

Through our analysis of the literature, we identify a set of interaction relations between decision agents, highlighting how their distinct roles operate in the process of combination. We introduce these as a novel set of contextual dimensions, which we propose delineate a space of combination.

Our awareness of this interaction space allows us to unwrap and expose some significant factors in the design space that are critical for success in Human-AI decision-making in the real world.

## Background literature: the critical lens

In this section, we draw on relevant literature to articulate our critical perspective and inform our stance on the use of the term *combination* in the title, providing both justification and clarification for its application.

We review key writings from Human-Computer Interaction (HCI) that address the significance of situatedness in human-AI interaction. We re-visit some of the robotics literature to draw out implications for (and distinctions from) how humans can use machines in decision-making. Finally, we integrate contributions from decision theory, along with further HCI insights on unremarkable AI, organizing these critical insights for clarity, and to ensure that our use of *situatedness* is accessible to the reader.

To *situate* is to place something in context - to describe the circumstances that surround it. Thus, we use *situatedness* in a way that could be substituted with *contextualisation*. However, as will be seen later (in Section 2.2), the term *situatedness* has significant precedent in human-computer interaction.

### Disambiguations - putting combination in context

We want to put *combination* in context in two ways. First, we want to make a strong connection between our use of the two words *combination* and *situated* in this paper’s title. Second, we would like to distinguish between *combination* and other vocabulary that is used to describe how humans act jointly with machines.

Our perspective on *combination* in decision-making centres on situatedness. We emphasise the various ways in which the diverse roles and contributions of humans and algorithms come together. These ways include structured and strategic coordination, such as protocols, as well as more fluid approaches like dialogue, interactive exploration, and potentially unstructured, opportunistic moves towards consensus or convergence. Among these ways we can discern several critical dimensions which we will elaborate as participating agents, control relations, task overlap, temporal patterning, informational proximity, informational overlap, input influence, and output representation coverage.

Our focus on *how* joint action is enabled means we direct attention to situated particulars. In turn, this ensures that the limits and opportunities for each contribution can be appropriately recognised and navigated. This extends prior work from Zhang et al. (2021) who argue for a more complete design space in which the cognitive effort required of the human participant is aligned with what they can most readily bring to the situated task. It is inspired by works such as Cai et al. (2019a) in which the human user is provided with interactive refinement techniques that serve a dual function - enabling task progress and increasing transparency. In another direction it also extends the concept of Human-AI Collaboration Protocols (HAI-CP), as discussed by Cabitza et al. (2023) which elaborates on an integrated set of rules and policies that stipulate the use of AI tools by practitioners (the human decision-makers in specific work practices) to perform a certain task. As with Zhang et al. (2021), this can be done by setting a specific timing for the provision of AI advice relative to the human decision-making process (see *human-first* ‘Hound’ or *AI first* ‘Ram’ protocols) (Cabitza et al. 2023).

How does *combination* relate to ‘cooperation’? Schmidt and Bannon emphasise how ‘cooperation’ in CSCW is facilitated *between humans* by means of ‘articulation’ work with the support of computers (Schmidt and Bannon 1992). This examination of *humans working together* is distinct from an exploration of *computers and humans working together*. We want to emphasise how *combination* may be facilitated *between computers and humans* by means of analogous work to understand and respond to the various features of *combination*. In this way, we pick up one of Hornbaek’s ‘blind spots’ which is the consideration of ‘multi-party interaction’, which he exemplifies with human-robot interaction (Hornbæk and Oulasvirta 2017), but we identify a more fundamental requirement, that of interactions involving representations themselves - the human-computer relationship as it deals with combinations of such conceptual representations rather than combinations of physical actions. We will return to this idea in Section 2.4.

Is *combination* a pre-requisite for ‘hybrid intelligence’? Akata et al. (2020) define ‘hybrid intelligence’ as a *combination* of human and machine intelligence (our emphasis), where Dellermann et al. (2019b) had previously specified complementarity as a definitional feature. The distinction is subtle, with Dellerman et al foregrounding the motivation - being that something is gained in the process. In any event, it seems obvious that *combination* is a pre-requisite for complementarity and is therefore the more general term. We acknowledge that Dellermann et al. (2019b) refer to a ‘contextual dimension’ and Dellermann et al. (2019a) already speak of ‘generic design dimensions’ as being requirements when developing hybrid intelligence systems. These dimensions inevitably include ‘task characteristics’ and ‘human-AI interaction’. We will return to the synergies between these design dimensions and our dimensions of *combination*. For now, we note that the hybridity concept appears to be founded on ‘deliberate task allocation’ Dellermann et al. 2019b, p640 rather than anything based on interaction or exploration of what might be complementary.

The dimensions in the proposed Dellermann taxonomy are novel. They are largely based on an examination of machine-development concerns first and foremost (e.g., task characteristics such as recognition, prediction, reasoning and action). This work differs by extending human-AI interaction beyond humans training or updating the model. In particular, we specifically consider sociotechnical context as the space within which the human-machine combination operates.

In what follows, we preserve the distinction between human and machine by avoiding terms that risk anthropomorphising algorithms when used to describe them. Terms such as *partner*, *teammate*, *collaborator* and *cooperator* would take us prematurely into consideration of where human attributes are assumed to be transferred to artificial agents. Attributes such as *agency*, *intent*, and *accountability* can remain unproblematically and exclusively human, while we still carefully explore how the distinct contributions are brought together. In this sense, we will allow each to ‘*combine their complementary skills and capabilities to make the best use of [their] distinctive strengths*’ (Ramchurn et al. 2021). We also draw on Licklider’s vision of ‘man-machine symbiosis’ from the 1960s as a deeply integrated human-computer *combination*, significantly enhancing problem-solving and real-time processing capabilities, requiring *‘tighter coupling between man and machine’* (Licklider 1960).

Having distinguished *combination* from related concepts like interaction and cooperation, we next want to review how prior work on robotics informs our specific context and the way we approach a survey of studies.

### Situatedness and interaction

Situatedness is an essential feature of human social existence. Writing in 1985, anthropologist of science and technology, Lucy Suchman, drew computer scientists’ attention to the significance of situatedness and its influence on human action:

$^{\prime }[T]$he mutual intelligibility that we achieve in our everyday interactions ...is always the product of in situ, collaborative work.’ (Suchman 1985, p 123)

In writing about interaction between humans who work with machines, she told us much about what human socialisation brings to the interactions we experience and subsequent HCI research has been heavily informed by her work (Twidale et al. 1994).

Suchman’s work showed how actions diverge from initial plans as a result of the impingement of situation particulars. In doing so, it highlighted a critical process we need to attend to if we are to improve the human-machine combination. Interaction that supports the detection and addressing of differences in perspective - that is, interaction that facilitates convergence of perspective - is a pre-requisite for the effective combination of contributions:

‘[T]he face-to-face communication that supports that work is designed to maximize sensitivity to situation particulars, and includes resources for detecting and remedying troubles in understanding as part of its fundamental organization.’ Suchman 1985, p 123

This not only emphasises the situatedness of the work itself, but it also emphasises how mutual understanding, for humans, is framed by a context and achieved through iterative interaction. When humans engage in this sort of interaction with each other, it rarely produces complete mutual understanding. Rather, it produces a partial understanding that is highly specialised to the context, and thus more readily approaches sufficiency for the task in hand.

Whatever recognition can be achieved by a machine of human actions or outputs, it will be of limited value unless a context is also provided or designed in place:

‘[E]very occasion of human communication is embedded in, and makes use of, a taken for granted but mutually accessible world’ (Suchman 1985, p 123).

So, as long as a computational or algorithmic agent has a less-than-human capacity to deal with the details of the situation and cannot take for granted a world that is mutually accessible alongside us, we must very carefully design the types of interaction we can have with it. Convergence of human perspectives and preferences with representations of their equivalents as held by machines requires appropriate, situated information flows. This is a core property of the lens through which our perspective has been formed.

Designs can incorporate the context for meaningful interaction if there is sufficient awareness in the design process of both the user’s objective and something of their interaction needs and interests. Cai et al. (2019a) provide an excellent example of this with an approach that uses insight from co-creation work and iterative design to create user-friendly operational levers that begin to align tractable representations that become mutually accessible by the human user and by the algorithm.

Our introduction pointed to the obvious problem facing any attempt to model human-computer interaction on human-human interaction - the challenge of creating mutually tractable representations (Wilson et al. 2023). To penetrate further into this challenge, we continue shaping our critical lens with a recognition that computer science is representational. It creates and manipulates models of reality, people, and action. ‘Computation is fundamentally a representational medium’ (Dourish 2001, p20). But Dourish quickly added an interactional qualification:

‘[A]s we attempt to expand the ways in which we interact with computation, we need to pay attention to the duality of representation and participation.’ (Dourish 2001, p20)

Duality is evident throughout Dourish’s argument. There is a tension between ideas and actions, between abstractions and concretions, between objectives and affordances. Dourish’s call for a fundamental shift of focus was aimed at seeking how human-centred interaction could be served, asserting that he was ‘more interested in interaction than with interfaces.’ (Dourish 2001, p3). This perspective directs our attention to the many and varied contextual constraints and opportunities for interaction - the space over which interaction must be considered. Our framework of dimensions begins to map this space.

Because we are considering artificial intelligence, however, we must address another potential ambiguity that arises from noting the situatedness of cognition itself. Any reference to Dourish in the context of AI needs to be clear on what is meant by ‘embodiment’. To clarify this, we take a brief detour.

Writing shortly after Dourish’s seminal work on interaction, Anderson (2003) reviewed how cognition among AI researchers was being re-considered as a situated activity and therefore how ‘embodied cognition’ should influence research. Smith and Gasser (2005) soon afterwards called for ‘embodied cognition’ in artificial agents so that they could experience and learn in some human-like way from their experience of the world. By extension, ‘embodied interaction’ has frequently been used to mean interaction with some physical object other than a recognisable computer in which computational technology is itself embodied.

Dourish’s view of embodiment, however, was that it was an approach “oriented toward the way in which *people* interact with systems" (Dourish 2001, p145 emphasis added). ‘Embodiment’, for Dourish, is a form of “participative status" (Dourish 2001, p18) that focuses on the human experience rather than the bodily form of the technology. Dourish himself has made clear that ‘embodied interaction’ was not intended to make ‘*a distinction between those forms of interaction that are “embodied” and those forms that are not*’ (Dourish 2013, p2:2). In exploring the meanings we develop through situated action, Dourish’s embodiment emphasises ontology, intersubjectivity and, above all, intentionality (Dourish 2001, pp128-138). Indeed, his view of embodiment foregrounds meaning and coupling (Dourish 2001, ch5). Thus, in setting up his foundations of interaction design, Dourish placed human experience at the centre of what he meant by ‘embodiment’:

‘Instead of drawing on artifacts in the everyday world, [embodiment] draws on the way the everyday world works or, perhaps more accurately, the ways we experience the everyday world.’ (Dourish 2001, p17 emphasis in original).

In this way, he was using embodiment to focus on the situatedness of human experience, not on the embodied design of the computer.

Our minor detour has helped further emphasise the significance of human situated action (i.e., participation) for the design of effective algorithmic systems. We explored the relevance of robotics research in an earlier Section 2.4. Here we want to emphasise the participative component of interaction in the context of decision support.

Interaction as an opportunity for convergence is central to how this research evolved and is presented. It has driven us to explore how prior work has addressed human combination with algorithmic systems for decision-making. In the situated use of computational tools, we must not only attend to the process we are attempting to support, but also to how workers, co-workers, creators and co-creators relate to this process. We need also to attend to the wider sociotechnical context and the effect of both shared and unshared objectives and capabilities between all these participants.

### Bench vs situated studies

We have emphasised the significance of situatedness as a critical factor in design and evaluation. Later, we will point out that situatedness is not a binary property of studies with which we can easily distinguish the situated from the un-situated. It might therefore seem counter-intuitive that we now want to justify the introduction of exactly that binary distinction. We do so as a means of highlighting what is revealed in our survey of decision support algorithms.

As with design, situatedness is a critical factor in evaluation, influencing whether solutions that seem effective in idealised conditions can be successfully translated into real-world clinical settings.

The specific distinguishing feature of evaluation that can predict effective translation is whether humans are asked to respond to the tool and hence whether the combination can be judged effective. As Ganguli et al. (2020) points out, it is what we observe about their effect in actual use that matters, not what we can say about the tools themselves. Indeed, clinically informed commentators have noted that the *AI chasm* described in Section 1 above may be an expression of there being ‘insufficient attention given to the factors that affect the interaction with [AI’s] human users’ (Vasey et al. 2021, p186). A 2022 editorial in *npj Digital Medicine* argued that ‘crossing the chasm’ requires improvement to both implementation and evaluation of AI (Marwaha and Kvedar 2022). Another opinion piece in *JAMA Health Forum* makes the point that while there is a lot of research on AI algorithms themselves, there is a dearth of evidence about how the real process - which involves the human-algorithm combination - performs (Elmore and Lee 2022). What we see in these commentaries from the literature is that few studies have incorporated situated evaluation of the algorithmic system on which they report. As a result, these studies have missed the opportunity to learn from situated users and there is no progress on what Cabitza and Natali (2022) call ‘adjunction’.

We attempt with our elaboration of dimensions of combination to ‘focus on the process-oriented and relational aspects of the joint action of humans and machines working together’ (Cabitza et al. 2022, p2).

Early in 2021, the process of developing new guidelines was announced with the aim of bridging the development-to-implementation gap in clinical artificial intelligence. DECIDE-AI (Developmental and Exploratory Clinical Investigation of Decision support systems based on Artificial Intelligence) invited contributions in an open and transparent Delphi process to reach expert consensus. In May 2022 the consensus was published in *Nature Medicine* (Vasey et al. 2022).

Many commentaries distinguish between *pre-clinical* and *clinical* studies, yet little is said about the evolution that needs to take place in pre-clinical work in readiness to make the transition.

The new guidelines refer to *in-silico* evaluation for pre-clinical studies and introduce *shadow mode* (or *offline mode*) as an intermediate evaluation mode between pre-clinical studies and live, large-scale clinical trials.

In this *shadow mode* the algorithm is situated in a workflow and allowed to make its suggestions, which can be logged but not acted upon. These suggestions are made in parallel with, but remain unseen by, human clinicians who make their decisions independently of the algorithm. This approach is sometimes referred to as *dual-running*.

Data are generated from both sides that show the difference between the machine suggestion and the human decision on the same case, producing very valuable results on prospective data. Still, this does not address the issue of evaluating the human-machine combination.

For this, we need what Zajac et al. (2023) call “iterative co-configuration and near-live and real-world experimentation". They call for more work to be put into addressing technical and social challenges of implementation since, without such situated evaluation, studies will continue to primarily measure things about the tool itself rather than its effect in use. The classic ML performance metrics obtained in the computer lab, such as accuracy, F1 score, sensitivity, specificity, etc. are critically important and necessary, but they are not sufficient.

We call these studies that confine themselves to an idealised operating environment *bench* studies, and we argue for greater recognition of their main limitation - that their results are often hard to replicate in a clinical context.

In *bench* studies, performance metrics are obtained in an artificial operating context where the relationships between humans, algorithms, and tasks are assumed. Performance metrics are often generated on pre-curated data inputs that may only partially reflect the features of real-world data. Although informative and necessary, these measures have proved insufficient as they frequently fail to translate into the world beyond the *bench* (Keane and Topol 2018; Cabitza et al. 2020; Ganguli et al. 2020; Liu et al. 2019; Vasey et al. 2021; Roberts et al. 2021; Laak et al. 2021; Marwaha and Kvedar 2022).

In order to make an appropriate distinction between the levels of ecological validity obtained in bench studies and those obtained in more realistic settings, we need to draw a sharp contrast between *bench* studies and the types of situated evaluation that take algorithms closer to clinical practice. We draw our binary distinction, then, between those studies that evaluate the human-machine combination in some way that attempts to replicate the eventual working conditions, and those studies that miss either or both of these elements of situated evaluation. Situated studies improve on *bench* studies because they evaluate systems by measuring variables, interactions and impact in more realistic operating contexts. This is particularly important when considering how clinicians and automation are to be combined. Cabitza et al. (2017) warn of a situation where machine learning places a focus on captured data at the expense of clinical context, which might not be represented. The combination, if it is facilitated, can help to mitigate such risks.

Ash et al. (2004) place healthcare information technologies as a whole under scrutiny, with a critique of systems that fail to align with their operational context. Systems that are not suitable for highly interruptive contexts, that ignore contextual relevancy in mandating interaction steps, or that impose fragmentation of natural workflows. This distinction echoes that made by Ala-Luopa et al. (2024) between ‘rational’ and ‘empirical’ approaches to AI design and development. Situated studies evaluate this requirement that technologies work in the places they are needed.

As mentioned, situatedness is not an identifiably binary property of studies but rather expresses a degree of ecological fidelity that ranges from basic inclusion of some realistic user conditions to full immersion in real-world operating environments. Our heuristic is to recognise any concession towards real-world operation as moving a study away from the bench. However, this must be accompanied by evaluation of the human-machine combination, and not the machine output alone.

The most informative situated evaluation for a given study will depend on the use-case. But it is likely to be one that involves the real end users. This is because no amount of documented requirements can capture the reality of working in a real clinical environment. Nonetheless, clinician time is a valuable resource. So designers of studies should seek to incorporate and learn from realistic conditions at an appropriate level early on, so that later evaluations can make best use of clinical time. This means incorporating situatedness into evaluations much earlier in the development process - formative situated evaluation. If we want the human-machine combination to work well, we must design for the combination. And we must evaluate the combination.

### Control relations in previous work

One of our dimensions (*control relations*) draws attention to how control is exerted. Our review of the literature reveals both similarities and distinct aspects in our view of this dimension. Robotics has long faced a related problem described as levels of automation (Sheridan et al. 1978), which involves a greater or lesser degree of allocating defined tasks to machines. A subsequent shift focused attention on joint execution of a single task step - what Johnson et al. (2011) called intra-activity dependence rather than inter-activity dependence. This new framing of interdependence *within* a task begins to outline a peer space.

In the call for *coactive design*, Johnson et al. (2011, 2014) target this space of joint action. However, there is a natural emphasis on ergonomic objectives, and this exposes a perspectival difference between robotic systems and decision systems. The concerns of observing, predicting and directing within a physical action space (Johnson et al. 2014) dominate over consideration of the abstract space of decision-making. Whereas interdependence in the decision space is informational and communicational, not based on conflict over spatial awareness, shared resources or task hand-off.

Johnson, Bradshaw and colleagues have come to recognise that joint action processes ‘are necessarily incremental, subject to negotiation, and forever tentative.’ Bradshaw et al. (2004, p22). This can be seen as a ready analogue to Suchman ’s (1985) recognition of the need for dialogue for ‘detecting and remedying troubles in understanding’, which, as we noted above, aims to approach understanding sufficient for the task through iterative interaction (see Section 2.2). It is our contention that this aspect must receive greater emphasis - the process of supporting convergence through iterative exchange. But while this facilitates control relations, it is distinct from them.

In reviewing Johnson et al. (2014), we acknowledge that a form of observability impinges just as much on the abstract decision space. Convergence here also requires exchange. But our emphasis is on the challenge of making this bi-directional with mutually tractable representations. Lai and Tan (2019) pick up the concept of a spectrum of control using much the same language as Sheridan, but they define their spectrum with specific machine behaviours where increased automation involves more information flowing from the machine to the human in the form of suggestion, explanation and confidence calculations.

There are reasons to suppose that each of these components and their tractable representations - suggestion, explanation, confidence - should be assumed to be an independent factor in how human-machine combination might be achieved (Jacobs et al. 2021a). Each is also distinct from, although influential in, the dimension of control relations.

Our dimension of control relations recognises that influence is under-studied in the context of clinical decision-making. We highlight the lack of material on combinations within the peer space (see Section 4.1. We recognise the debt our concept of the dimension of control relations owes to these previous authors. But we argue that any usage of the dimension of control relations in situated decision-making is distinct from these contributions and therefore novel.

### Decision-making as situated action

The process of combined decision-making can be seen under three headings: *Modelling* the specifics of the decision task (e.g. prediction and classification); *Exchanging* information within the space (e.g. interaction and visualisation) to promote convergence between agents; and collectively *Resolving* the task by concluding the decision (e.g. by sequential decisions and/or consensus or elective process). Having recognised the processes involved in combined decision-making, we need to go deeper to unpack what constitutes Suchman’s ‘mutually accessible world’, the ‘objectives’ already oriented to, the relationships between agents and the constraints of exchange.

Despite some fundamental divergences from Suchman, the writings of Winograd and Flores give us an allied insight into what is beneath the surface in human decision-making. They describe a key process we employ in situated decision-making: “The principal characteristic of deliberation is that it is a kind of conversation... guided by questions concerning how actions should be directed.” (Winograd et al. 1986, p149). This allows for the kinds of adjustments and interactions that we must consider as soon as we consider combined decision-making.

And there is useful background literature on decision-making that can further shape our critical lens and help us anticipate how the framework dimensions we will elaborate in the next section arise from decision-making theory as much as from HCI.

Group decision theory emphasises that greater consensus is best achieved through dialogue and interaction ahead of a formal resolution - the convergence of *preferences* prior to a group decision (Cheng and Deek 2012). Multi-agent groups have diverse value-perspectives that break the assumptions of classical decision-theoretic methods (Thornton et al. 1992; Howard 1968).

Social Choice Theory shows that convergence of *results* can often be achieved through iteration of the elective process (Lev and Rosenschein 2012), a process necessary in multi-agent groups to avoid the default bias of supermajority rules.

Whatever the analogues of preferences and value-perspectives encoded in machines, there is still an argument that we should recognise how these are interacting with human opinions and that convergence is possible through iterative exchange. At the same time, any version of an elective process is a compromise (Satterthwaite 1975; Gavish and Gerdes 1997; Chatterjee 2017).

These recognitions impel us to consider two contextual dimensions of combination: the multiplicity of *participating agents*, and the *control relations* of these agents.

Decision analysis can help us decide between treating a compound set of steps as a single decision and considering individual steps as distinct decisions. If the influences that inform and condition each step are unchanged at each step, then they can be considered as a single decision (Howard and Matheson 2005). Conversely, if informational or conditioning influences change between two successive steps, then we can no longer compound them into a single decision. A further special case arises if the outcome of one decision is an exclusive influence on another. A common clinical arrangement is to have a pair of successive decisions that appear to consider the same question. This first step is frequently called a triage decision - its purpose might be to enrich the onward case mix with high-priority instances, or filter out low-priority instances to reduce the total volume. Accordingly, it might be beneficial for a preliminary decision to have high specificity or high sensitivity compared to the subsequent step. Where the two decision events process exactly the same input information, the two together can be considered a single decision, but often this kind of workflow is designed specifically to enable the downstream step to access and consider more detailed information. An inevitable trade-off is usually a feature here. A high volume of decisions on relatively scant data at the first step allows more effort per case to be devoted to more gathered data on fewer cases in a subsequent step. Where the informational influences are distinct between the two steps in this way, or where the decision-agent value preferences are not identical - such as when cases are passed on to a distinct process, then decision analysis makes clear we should avoid treating them as if they are a single, compounded decision. This distinction has important implications for how we analyse and adjust when trying to improve decisions from such compounded steps. We can clearly see the process of resolving here, however, varying influences upon a compound set of steps hints at two further contextual dimensions - The contribution of multiple agents on a given task, *task overlap*, and the enacted contribution across time, *temporal patterning*.

A further adjustment of this critical lens is worth making with reference to a phenomenon understood by designers of good computational tools. And again, we take the opportunity to point out the influence of this literature on our framework of dimensions.

As well as providing immediate affordances, technology in ‘real-life contexts’ inevitably includes indirect affordances through mediation processes such as aggregation (Kaptelinin and Nardi 2012). Working effectively with a busy individual or team of humans will frequently require some level of *aggregation affordance* from a system. Combine this with the powerful observation in the HCI literature that ‘Good tools enhance invisibility.’ (Weiser 1994) and we begin to see how much an effective design relies on anticipating its situated use. Marc Weiser’s description of how effective tools allow you to see the task and to observe your progress while sublimating any attention the tool itself might get gave rise to the powerful concepts of *unremarkable computing* (Tolmie et al. 2002; Yang et al. 2019). This is particularly important in systems providing decision support to domain experts.

The property-set of availability and usefulness with non-distraction is a strong requirement in real clinical contexts. New tools need to fit into existing workflows (Sendak et al. 2020a; Lee et al. 2021) and assist rather than introduce disruption. Compared to more typical human roles, those who work in medicine are relatively comfortable with lots of information, being necessarily highly tuned to information-relevance. To provide useful support, their computer systems need to be equally discriminating about when they draw attention, akin to a human team member. There must be a recognition that ‘humans and machines are embedded in complex organisational and social systems’ (Shneiderman 2020), and that solutions must attend to these same systems (Suchman 1985; Hartswood et al. 2003). Two further contextual dimensions emerge here: the availability and situated nature of information related to decision making, *informational proximity*, and the shared space of relevant information, *informational overlap*.

In sociotechnical systems, established sequences of decisions can exhibit even more complexity and can involve both positive and negative interaction effects. Knowing something of how a decision output of their own will be regarded and responded to by someone (or something) else may influence how a human’s decision is made. A simple example would be if a doctor marks a referral for heart bypass surgery as an emergency procedure because she knows that it will expedite the procedure in a context where an imposed budgetary quota for non-emergency referrals for the period has been reached (Hunter 2007). This gaming of a process inevitably alters the combined effect of a sequence of decisions, and even mere suspicion of the gaming action can lead to compensating behaviours in other parts of the sequence. Only rigorous situated evaluation can reveal the combined effect. These effects of decision-making give rise to two further dimensions, *input influence* (intended or otherwise), and the presentation valence of outputs, *output representation*.

### From background to survey

This analytical perspective, grounded in the literature, informs our review of individual papers in the survey. We use insights from the commentaries to guide our focus on relevant theory. We also draw from specific algorithm studies to exemplify the contextual tasks, moving well beyond the classic prediction and classification tasks beloved of computer science. This theory section (Section 2) informs, and is also informed by, our exploration of situated studies to ensure consideration of the real combination space inhabited by humans and machines. It is on this situated evidence that we base our argument that the ‘space of combination’ is under-explored. To engage with the factors that shape combination, we have to focus solely on situated studies. What we can see in reviewing these studies together are the gains made from attending to interdisciplinarity, co-creation and formative evaluation as well as how these gains are evidenced in situated and prospective evaluation.

## Survey method

This paper is aimed at clinical innovators and AI modellers as well as practitioners in Human Computer Interaction (HCI) - those who recognise the improvements that can be made to advance our collective contribution to the field of improving clinical outcomes. In surveying the literature, our objective is to examine papers from sources that are closest to making a difference in a real-world context.

Before we begin, we should note that decision-making is not the only clinical task that is set to be within reach of algorithmic influences. Robotic-assisted surgery, and deep-learning methods for knowledge discovery in fields such as genomics and proteomics are areas that are likely to be impacted greatly by the further development of machine learning (Esteva et al. 2019; Jumper et al. 2021). But we leave these areas aside as distinct from specifically clinical decision-making. There is also a fast-growing field of research on large language models (LLMs) that promise new and persuasive forms of decision-making assistance (Singhal et al. 2023a, b). However, at present there is little research on the situated use of LLMs to support decision making as part of a clinical workflow. We also do not aim to touch on algorithmic resource management such as scheduling, rostering, costing or medicines management. Our focus is on the kinds of decisions that clinicians (and their patients) make routinely in the course of clinical care.

**Fig. 1:**
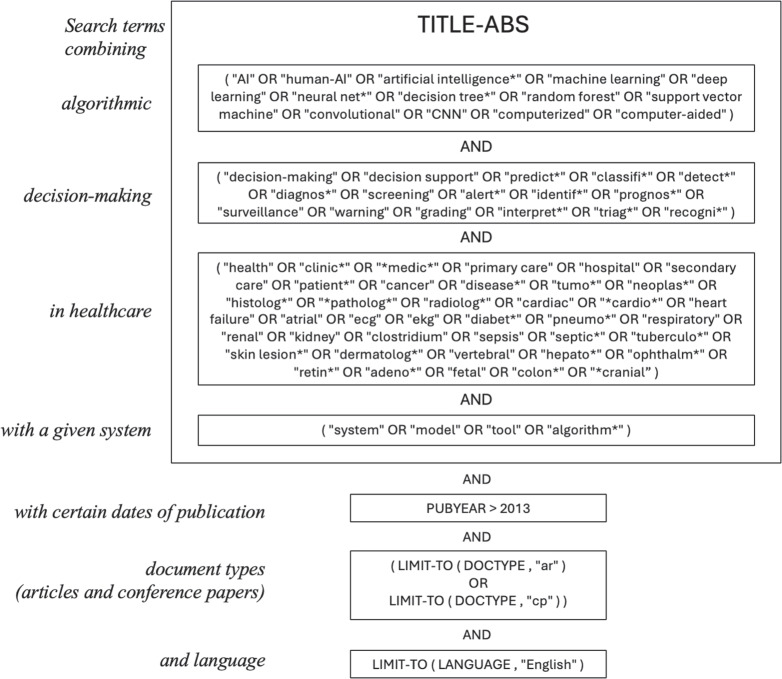
The search query used for initialisation with Scopus (https://www.scopus.com).

We performed a scoping search seeking papers reporting on algorithmic decision support systems for clinical tasks. The scoping search showed that the search criteria were complex, and that there was a significant rise in papers of interest after 2013. A structured query (see Figure 1) of the Scopus database yielded 102,000 records. To provide an initialisation for manual screening, we selected the top 350 papers based on relevance and an additional 350 based on citation count. Together, these formed the input for a screening step focused on identifying work related to clinical, human-machine combination, which resulted in 203 papers (a 30% hit rate). A more detailed review of these papers was followed by an enrichment process, using citations from the selected papers and key authors, which added 145 more relevant and authoritatively cited papers. This brought the total to 348 papers, all of which were then examined in a full-text review.

**Fig. 2:**
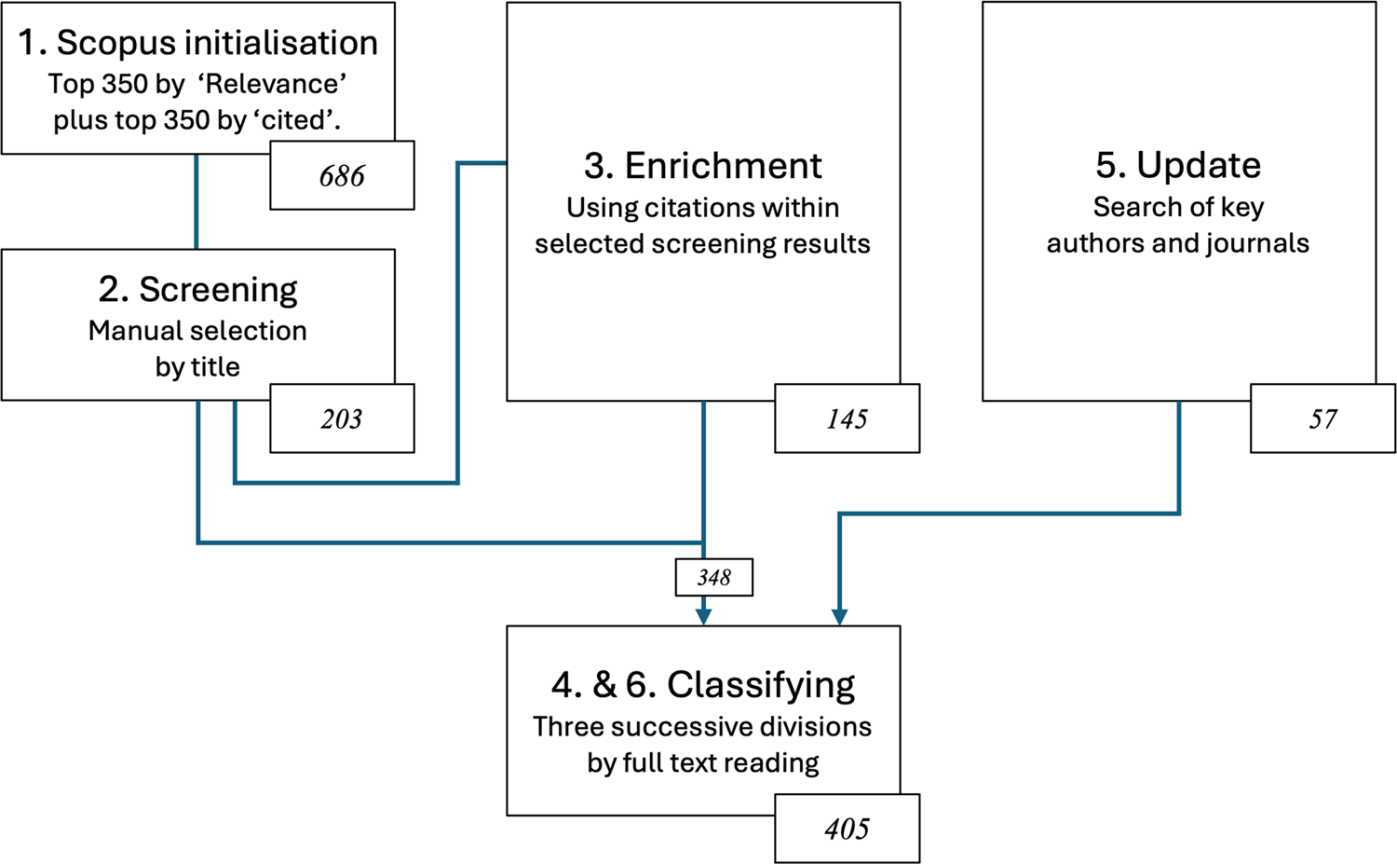
The search query used for initialisation with Scopus (https://www.scopus.com).

The process is illustrated in Figure 2.

**Fig. 3:**
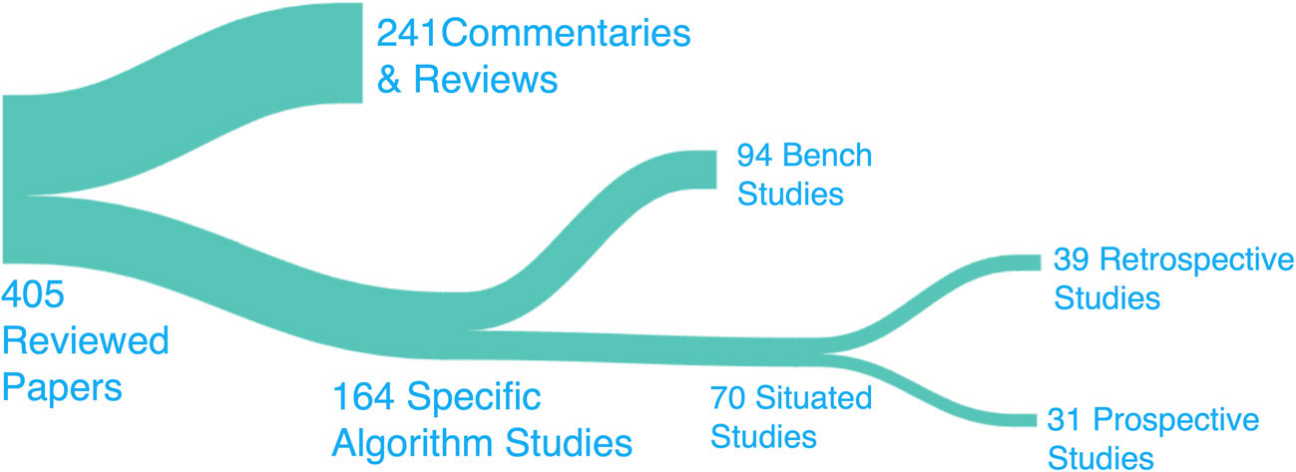
Types of paper in our survey. Terms are described in the text.

We classified this tranche of material by means of three successive sorting steps, seeking to establish the relationship between the literature and meaningful situated evaluation. The four resulting classes appear in Figure 3, which shows the final version of this process. In the first step we sought to separate those reporting specific algorithm studies from broader commentaries and reviews of the topic. Then, for the algorithm studies, we separated the situated studies from those conducted in a more idealised setting - *bench* studies. Finally, for the situated studies, we distinguished between those gathering retrospective and prospective evidence of performance.

We observed that our search yielded a large number of commentaries and reviews, reflecting our interest in using digests of related studies as an efficient way to identify situated studies. This also highlights the significant level of interest in research and analysis within this rapidly evolving field.

A subsequent update search of key authors, journals and their references yielded an additional 57 papers. These were put through the same classification process, bringing the total to 405 as shown in Figure 3.

Distinguishing papers that offer a commentary or review of a particular setting or technology and those that evaluate a specific algorithm is relatively straightforward. Papers that include an evaluation of a specific algorithm are included in the latter group.

Fortunately, distinguishing between bench and situated studies is also straightforward in many cases. An archetypal bench study describes only the algorithm and the single task it supports, without going into detail about the evaluation setting. Frequently, the test dataset is reported as being a standard, published resource. At the other extreme, a recognisable situated study reports the clinical institution and some facts about the specific setting or arrangements providing for study conditions. However, challenges arise in cases where evaluation takes place in a clinical institution, but descriptions of the workflow and specific environment are left unreported. As already mentioned in Section 2.3, situatedness is not a binary property. So classification relies on establishing a threshold of contextual relevance. In these situations, we examined the results and discussion sections for details and clues revealing the presence of real-world factors that do not impinge on the typical bench study.

To separate prospective from retrospective studies is usually a little easier. The simplest indicator is explicit mention of the word *prospective* in relation to test cases. In cases that appear ambiguous, it is sometimes necessary to examine the timeline descriptors to establish whether a study used algorithm input data from prospectively captured cases.

In the resulting classification, as noted, a large number of papers were commentaries or reviews. Just over 40% of the total represented algorithm studies, with over 74% of the latter (121/164) originating from clinical journals. The remaining quarter comprised papers from bio-informatics, computer science, HCI and general academic journals. Bench studies slightly outnumbered situated studies, with the initial division heavily skewed toward bench studies after our first pass.

However, enrichment and author searches allowed us to more closely target situated studies. Similarly, with retrospective and prospective data collection, the numbers for the retrospective class were far greater than the prospective class on our first pass. In the enrichment step, we specifically sought out references indicating prospective studies.

The overall process used was necessarily explorative and was aimed at discovering the relationships between these different approaches to algorithm evaluation. It was fluid, iterative, convergent and content-driven in order to uncover a landscape that has not previously been mapped out - in search of features that have not elsewhere been analysed. The sampling and enrichment approach allowed us to use the commentaries and reviews alongside the specific studies to provide pointers to a larger proportion of situated and prospective studies than could be pinpointed by any search queries.

A specific limitation of our approach is the absence of a formula or structured query for the enrichment process we have employed. In that sense, we prioritised iterative, content-driven convergence over reproducibility. However, our experience suggests that manual screening by title from a larger initialisation set is neither efficient nor reliable - nor is it a reproducible method for identifying examples of the situated evaluation we seek to examine.

Our examination of the space shows that, despite an attempt to search specifically for situated studies among papers reporting on specific algorithms, we found a larger proportion of bench studies. And the count of retrospective studies outweighed that of the prospective. We conclude that situated and prospective studies are harder to find because they are fewer in number in the sampled population.

After enrichment, a majority of the algorithm-specific studies were found to focus on de-contextualised *bench* performance rather than situated evaluation. Situated studies (70 papers) that begin to reach across the translation gap have less than half (31 papers) being prospective studies (those making use of new data), which are the studies that produce the best empirical evidence. Although not a systematic review, it is striking that less than a tenth of all the retrieved papers report prospective situated studies.

## Eight contextual dimensions of combination

The *AI chasm* is a recognisable result of the lack of situated evaluation. This gap seems to stem from the separation between algorithm development and HCI as distinct disciplines. Simply gathering the outline requirements from an end user is not sufficient. We need development that incorporates their needs, interests and situated opportunities to interact with a system.

Combination requires interaction. So we have to recognise both aspects of the interaction duality - participation and representation (see Section 2.2) - and argue for the need for mutually tractable representations. And these require machine learning (ML) developers to actively involve the humans who will use their systems. It means exposing the algorithm’s features, capabilities and limitations during the design process and exploring which aspects benefit the user and determining what levers the user should have to effectively communicate their queries to the machine (see Cai et al. 2019a).

The interaction should be geared towards the concrete use-case. The closer it comes to supporting the iterative, incremental exploration of options, the more readily it will be able to do this.

If we want interaction that serves the process of convergence (see Section 2.5) and the decision-making version of the coactive approach, then we have to design systems capable of such interaction.

Our reading of the literature for this survey points to the need for situated co-design and early, formative situated evaluation as the best way to secure translation across the *AI chasm*.

What ML and AI developers need, then, is a way to incorporate situated design and anticipate situated evaluation. We believe the eight contextual dimensions of combination provide a framework for developers to move in this direction.

Each use-case will have its specific space of combination, with features that are more or less determined by a sociotechnical context. An appreciation of the possible features of the specific combination space equips the developer with a greater awareness of the significance of design decisions. While technical and resource constraints may impose certain limitations,

insufficient domain knowledge (or the lack of a suitable framework for seeking domain-specific information) can lead to decisions being be made without awareness of their downstream impact once the system faces a trial deployment in a situated, real-world context.

To derive our dimensions of combination, we therefore abstract from the concrete particulars of many decision situations what is common to them as a collection - their property of being instantiated in a sociotechnical workflow - the characteristics, constraints and opportunities of this workflow always display certain features in relation to task, timing, information, influence, participation and control. While we do not argue that this list is exhaustive, we believe the eight dimensions we elaborate and exemplify here serve to justify the claim that this is both a novel and useful framework.

In this section, our abstraction means we excavate beneath the more obvious features of decisions (their options and their outcomes) and look at the underlying relational factors that inevitably arise from their sociotechnical context. From this perspective, it becomes clear that the way distinct contributions are brought together introduces multiple contextual dimensions to the combination space.

In unpicking these *contextual dimensions of combination*, we depart from the bulk of previous literature. This paper draws attention precisely to what is distinctive about the situatedness of different contexts for Human-Machine combined decision-making in a clinical context, a distinctiveness we can only illustrate with situated studies since these, unlike bench studies, provide detail about the context in which the human-AI combination operates. We describe these dimensions as the *contextual dimensions of combination*, and give pointers to how these contexts might be noted together.

**Fig. 4:**
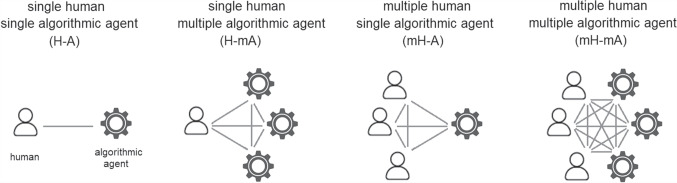
Dimension of participating agents.

**Fig. 5:**
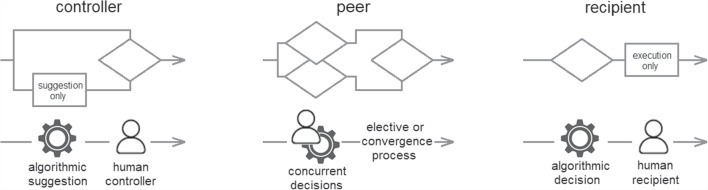
Dimension of control relations.

**Fig. 6:**
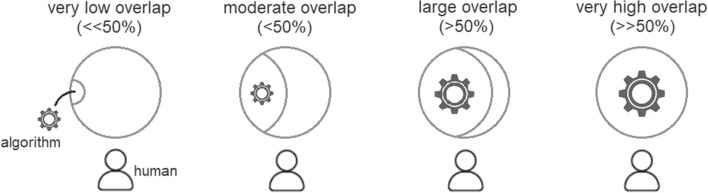
Dimension of task overlap.

**Fig. 7:**
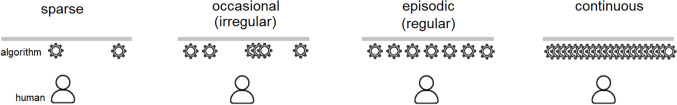
Dimension of temporal patterning.

**Fig. 8:**
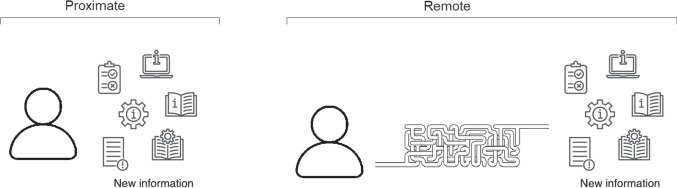
Dimension of informational proximity.

**Fig. 9:**
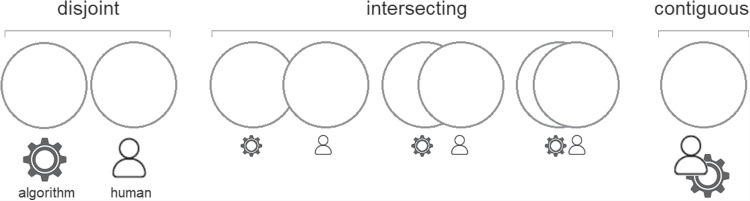
Dimension of informational overlap.

**Fig. 10:**
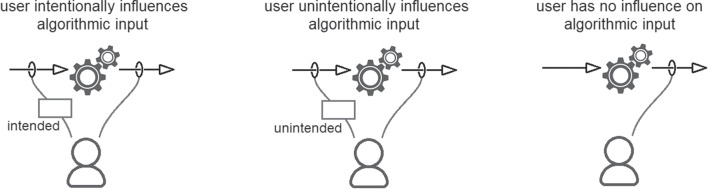
Dimension of input influence.

**Fig. 11:**
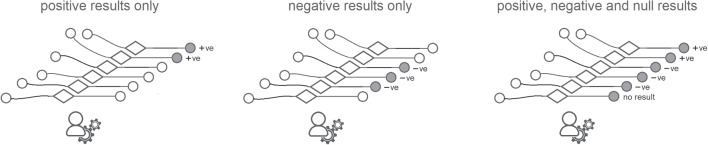
Dimension of output representation coverage.

The eight contextual dimensions of combination listed below were inspired by theory in the previous section and refined through iterative reading and analysis of the literature presented in this section. We elaborate their significance in the next section, here we briefly introduce each one.

- The *participating agents* dimension (Figure 4) expresses how the numbers and types of agent combine - How many humans with how many algorithmic decision agents.
- *Control relations* (Figure 5) determine whether a human participant is expected to override or authorise decisions suggested by an algorithm or is simply intended to accept them as recipient - or, indeed, whether each agent is a peer-participant in the process.
- *Task overlap* (Figure 6) accounts for how much of an agent’s work is supported by the combination - it may be a high or low proportion.
- *Temporal patterning* (Figure 7) is a description of how the combination is enacted over time - whether occasionally, episodically or continuously.
- *Informational proximity* (Figure 8) primarily concerns information access, indicating how readily an agent can acquire new or additional information relating to a decision.
- *Informational overlap* (Figure 9) expresses how much the participating agents share access to immediately available information.
- *Input influence* (Figure 10) distinguishes between arrangements where users have an intentional influence on algorithmic inputs, an unintentional influence or no influence at all.
- *Output representation coverage* (Figure 11) tells us whether positive, negative or all possible output values are produced - whether there is full or partial coverage of output representations.

While each of these dimensions can be seen as basic, each one adds complexity and challenge, and the space is rapidly sub-divided into many different *situated types*. In the following sections, we define, exemplify and highlight the insights these bring to combined Human-machine system design.

### Participating agents and control relations

The *participating agents* dimension (Figure 4) expresses how the numbers and types of agent combine. The number and type of participating agents clearly makes a difference to any formalised process of deliberation, but also impacts the process of sharing information and attempting a convergence of perspective which is critical for leveraging different insights. At one extreme on this dimension we see a single human operating with a single computer algorithm (the classical dyad of human-computer interaction). And at the opposite extreme are situations where multiple humans operate with multiple participating computer algorithms. There are plenty of examples of the former (85% of situated studies report on systems that are dyadic) - but no instances that we found, currently, of the latter (multiples of each agent-type) - although we speculate the prospect may not be too far off. We locate four distinguishable types on this dimension (Figure 4):

- *H-A* (single human - single agent) - A single human working with a single algorithmic agent in the classic dyad often assumed as the template for human-computer interaction.
- *H-mA* (single human - multiple agent) - A single human receiving decision support from multiple algorithmic agents. The human experience here is distinct from working with a single algorithmic agent.
- *mH-A* (multiple human - single agent) - A single algorithm providing decision support to more than one human. The humans in this situation may be dispersed or co-located and their resultant action may be individual or as a group.
- *mH-mA* (multiple human - multiple agent) - Combined decision making between multiple algorithms and multiple humans. Assembling the complexities of multiples of both types.

*Control relations* (Figure 5) determine whether a human participant is expected to determine an algorithmically supported decision, merely receive the output ready made by the machine, or something in between. In control relations, there is a conceptual connection to governance, which Katzenbach defines as ‘coordination between actors based on rules’ (Katzenbach and Ulbricht 2019). But these are not machine rules. They are the, often unwritten, expectations of where control lies in a decision-making situation. Therefore, real-world practice is more complex and nuanced than may first be conceived by a system designer. As Katzenbach points out, there is rarely a binary distinction between a human being in or out of a loop. Rather, there exists in most real cases a control spectrum.

In robotics (see Section 2.4) we encounter little confusion from the use of terms like *control*. But in the context of human-AI combination for decision-making, an attempt to describe this spectrum and the variability of control and autonomy must begin by making clear whose control or autonomy is being described. What is being controlled is not a physical object such as a machine part, an item on a production line or warehouse package. Control of a decision is rarely directly observable. In this discussion, we (arbitrarily) choose to speak of human control and contrast it with algorithmic autonomy. Work to describe this spectrum outside of robotics has been done by Saetra in relation to political decision-making (Sætra 2021). Saetra proposes that AI’s role in decision-making processes can be conceptualized as six functions: to support, assist, augment, alleviate, automate or supplant human decision-makers. Specifically, and relevant to our discussion, these functions are not intended to describe preparatory algorithmic processes such as information gathering, data analysis, or automated suggestion-forming. More importantly, they highlight the various roles an algorithm can assume, depending on its degree of autonomy in relation to the user. These roles range from maximal human control in the ‘Human-in-Command’ scenario, where AI serves purely as a *support* tool with neither initiative nor autonomy, to the ‘Human-out-of-the-Loop’ context, where the level of human involvement is limited to receiving the result, effectively *supplanting* human decision-making.

There are situated clinical contexts where this latter arrangement is conceivable. An example below (Section 4.1.1) describes an impressive study in which the algorithm user controls only in the sense of positioning the algorithmic device in relation to the patient’s eye and making an onward referral if one is indicated by the output. Humans capable of evaluating the resulting decisions are controlling with some degree of separation, by means of post-hoc auditing or sample monitoring.

As with participating agents, then, if we draw out the extrema, we see the dimension of *control* spanning between two poles - at one pole we have support from an algorithm that has little or no autonomy itself - at the other, there is automated decision-making where an algorithm has full autonomy. In between we find different degrees of human control and different levels of algorithmic autonomy.

We consider three different points on this dimension (Figure 5):

- *Human as controller* - The human acts as controller. The algorithm provides a suggested decision outcome and the human makes a subsequent decision on whether to accept this or reject it in favour of their own deliberation.
- *Human as peer* - Each agent provides their initial decision or inclination, and some form of elective or iterative convergence process determines how these are combined.
- *Human as recipient* - The human is merely a recipient of a decision made by the algorithmic agent. Algorithmic objectives completely determine the outcome. The human acts only to execute the algorithmic decision and does not participate in the decision-making process itself.

Each of our first two contextual dimensions presents interesting subtleties and allows us to divide the space into multiple *sub-spaces* (Table 1). The evident complexity, however, may yet hide the fact that there is actually a danger of over-simplification here. Quite apart from all the possible different numbers of agents involved, we should consider whether each might have a distinct role. A simplified classification like the one just provided implies that multiples of each type are homogeneous, but in reality they allow for mixed and uneven participation.

In multi-agent arrangements, subsets of agents may have their own elective processes to aggregate their preferences and make a contribution as if they were a single agent. And such subsets might vary in their constituents and in their commitment to internal aggregation. In these and many other ways, the contribution and influence of each could be quite different from others of the same type. A common configuration in healthcare is the triadic arrangement of a patient, their clinician and a computer (James et al. 2022) - a situation that needs to be highly sensitive to differences in information, awareness and preferences. Another is the Multidisciplinary Team Meeting (MDT) case discussion. Participants frequently have distinct roles, responsibilities and objectives so the inclusion of an algorithmic contributor (or of more than one) should be designed carefully, with the team dynamic taken into account.

While our roles of *controller* and *recipient* appear to be mutually exclusive, in practice, these roles can often end up being blurred. Automation bias can lead controllers to be uncritical recipients of algorithmic input (Skitka et al. 2000). And the related phenomenon of automation complacency can lead people and organisations to make less effort to monitor algorithmic processes for errors (Grissinger 2019). Conversely, if a human operative is unable to trust some algorithmic output that they are meant to merely passively accept, they end up controlling the contribution.

There are potential pitfalls in implementation too. For example, a human having the authority to override an algorithmic suggestion does not necessarily make for a smooth relationship if the process of asserting control requires a continual and unrelenting additional effort - e.g., the persistent, but unhelpful and disruptive, alert (Wong et al. 2021).

There is a further complication in the dimension of *control relations*. While our dimension describes the formal arrangements for where *control* lies, there is a distinct effect arising from the *influence* of machine suggestions. And it is known that these effects can be altered, for better or worse, by factors such as order effects and explanation. As mentioned in Section 2.1, if a human elects their initial outcome before a machine suggestion is revealed, the effect can be different from if a machine suggestion is made first (Cabitza et al. 2023; Green and Chen 2019). Moreover, the effect of explanations appears to be both context and user-dependent. For non-expert users, explanations are likely to be more persuasive than might be useful - thus impacting and possibly undermining the intended control relation (Alufaisan et al. 2021; Jacobs et al. 2021b; Cabitza et al. 2024). We currently propose neither an *elective sequencing* dimension nor a *persuasion* dimension in this framework, as the effects of these dimensions are not well evidenced in the situated studies we found. So, they are seen as good candidates for future work (see Section 6). It is clear, nevertheless, that careful situated evaluation should explore the possible impacts with realistic replication of timings, expertise and any explanation approach - and future work should aim to explore these effects more systematically.

To consider examples, we look only at situated studies. *Bench* studies, as described above, by definition, stipulate few, if any, of the details of their context of use - especially how and by whom an algorithmic output is used. The 70 situated studies (Table 1) are not evenly distributed in this representation of the situated space - the *contextual dimensions of combination*. They include only nine systems with a human acting as a recipient of algorithmic output. The majority of the studies (the 49 occupying the top row) cover systems in which the control relationship provides for a human acting as controller over the algorithmic output. Most studies (60 occupying the left column) report on systems that are dyadic (having two agents). No studies were found that involve human and algorithmic agents as peers in a decision process (middle row). The two groups, retrospective and prospective situated studies, show essentially the same distribution as each other - occupying the top and bottom rows and skewed toward the dyadic.

We should allow for attempts at peer-participation as a likely development that will fill out this combination space. We contend that system designs will increasingly occupy the vacant area in Table 1 as technological trends in ubiquitous computing, mobile devices, teleconferencing, voice interaction, language models, visualisation, tractable representation and multi-disciplinary team working progress. And work will consequently spread out from the current concentration in one corner of this space. It is therefore imperative that we become aware of the space as a whole in order to detect and accommodate this shift appropriately - a need we will return to in due course.

**Table 1:** Dimensions for ‘Participating agents’ and ‘Control relations’ and their frequency in the literature (NB. The adjective labels, ‘Controller’ etc, describe the role of *humans* in the combination).

		Participating agents			
		H-A	H-mA	mH-A	mH-mA
Ctrl relatns	Controller	41	1	7	
	Peer				
	Recipient	19		1	

#### Examples (Participating agents & control relations) from situated studies

We now take a look at some examples from situated studies through a new lens formed by our awareness of the *participating agents* and *control relations* dimensions.

Natarajan et al. (2019) present an example of dyadic combination in which the human is a non-specialist who serves to execute the referral decision of the algorithm. Of course, the decision is not one of treatment directly, but it is a consequential pathway decision. Cai et al. (2019a) provide a design example for a dyadic system where the human controller is able to access and explore different facets of the algorithmic contribution during an exploration phase that is analogous to the exchanging described in our critical lens above, and ahead of a final decision. The tools are the product of careful co-development with end users and iterative evaluation to test their real-world applicability.

Cai et al. (2019b) also provide some useful evidence of how appropriate on-boarding can help bolster effective use of AI systems by promoting convergence in this way.

Our example of a (non-dyadic) single-user, multiple-computer system is from De Fauw et al. (2018). This study shows multiple algorithms providing their distinct results to a human user who is the controller, deciding in response. The user can see (at a glance) the ‘preferences’ of each algorithm on diagnosis, effectively creating a form of staged voting - the human is able to see the degree of concurrence among the algorithms and hence is prompted to assess the strength of algorithmic opinion. The system is also notable for including ranked assessment of multiple pathologies - something that is immediately beneficial in practice when provided in a form that practitioners can access, interpret and trust.

In alert systems, such as reported by Sendak et al. (2020a) and Bansal et al. (2018), it is common to broadcast the alert signal to multiple users. However, many circumstances inevitably see one human agent consume a signal while others remain passive observers. The resulting action is only nominally a combination of multiple users with the algorithm, but a process of decision-making must be assumed to take place within all those who see the alert. So, organisational protocols and behaviours will be a much greater factor in how effective such a system is in practice. Details of the system’s interaction with the wider user group rapidly become insufficient for understanding how such a process succeeds or fails in improving care. In fact, in both these deployed systems a dedicated role is created to carry out the first step in a sequential process. The effectiveness of the whole then rests as much on the design of the workflow as on the individual steps, of which the algorithmic contribution is just one in a series. In each of these studies, time was invested in continual cycles of implementation and situated evaluation to find the optimal combination of sensitivity, specificity, efficiency and sustainability in the context of the human teams surrounding the system. Building the algorithm was essential, but it was a small part of a much bigger process.

On the other hand, a well-designed alert system for a given context can have wider sociotechnical effects, increasing user awareness of, and attention to, certain clinical factors. This can mean that the human role is enhanced beyond the interaction in a given case (Brocklehurst et al. 2017). The training effect as well as the awareness of other human agents as participants can have real effects (in diverse ways) on effective decisions and outcomes. The examples we have selected hint at the potential for systems to move into the empty parts of this *space of combination*. But the bulk of studies are still far from doing this, and no examples were found where humans and algorithms occupied a peer space. As mentioned, this represents a part of the combination space that is highly underdeveloped - but which is likely to be populated in the next period. And note that the complexities introduced by combining multiple humans (each potentially with different authority) and the possible separation and re-combination of humans are factors that might change during the use of a system.

It is notable that all examples of human recipient roles are from ophthalmology. This reflects both the maturity of image processing techniques and the incentive to address an increasing global prevalence of diabetes-related vision-threatening retinopathy by means of automatic risk prediction that indicates an appropriate clinical pathway. The big takeaway is that situated evaluation, especially when prospective, provides a clearer picture of clinical value.

We now turn to some dimensions that explore the realm of cognitive burden.

### Task overlap and temporal patterning

*Task overlap* is the extent to which two or more agents are occupied in common decision tasks as part of the total required decision tasks in a constrained period (Figure 6). In practice, for humans, it is the proportion of their task-set that is impacted by the contribution of the algorithm. At one extreme, there may be algorithmic support for a very small fraction of the human task. For example, an ambulance dispatcher has to consider which one out of tens of possible incident descriptors a call in progress should belong to. A system that gives a binary opinion on just one of these is supporting just a fraction of the human’s current cognitive burden. In such a case, the design may need to account for the human cognitive process of tuning-in to the algorithmic contribution. And the performance of the algorithm may itself need to be tuned-in to the fractional role it plays in the human’s task-set. At the other extreme, the algorithmic support may extend to cover the entirety of the human task-set, so that the contributions are contiguous. For example, a computer vision system that highlights adenomas during a bowel screening endoscopy could be supporting the entirety of the clinician’s task at that moment. We use points on the *task overlap* dimension to typify four types, as can be seen in Figure 6:

- *Very low (*$\ll$*50%)* - A minimal overlap between the algorithmic assistance and the current cognitive burden of the human.
- *Moderate (*<*50%)* - Less than half of the human’s current cognitive burden is assisted by the algorithmic contribution.
- *Large (*>*50%)* - A large task overlap means that most of what concerns the human at this moment is being supported by the algorithmic agent.
- *Very high (*$\gg$*50%)* - Effectively the task facing the human and that addressed by the algorithm are contiguous (they completely overlap).

*Temporal patterning* describes how inputs are combined over time. Quite often, a human task or set of tasks is supported by an algorithmic contribution that is accessed when needed at the discretion of the user. A user may invoke some help function containing the assistance. Or the interaction may be instigated by the algorithmic agent. For example, an intelligent alert may call the human’s attention to a developing problem. Either way, the timing of successive periods of combination can be sparse or intermittent in the human workflow. Or it can be continuous. A diagnostic assistant might be invoked on only a proportion of new patient consultations during an outpatient clinic, producing a sparse patterning. A routine ophthalmology screening clinic might make use of the same eye scan in every appointment, producing an intermittent patterning. An intelligent endoscopy video system may not only be running continuously during the procedure, but may be providing a continuous support to the endoscopist. Note that if the algorithmic model only engages with a human user intermittently, even if the algorithm itself is running continuously, then the temporal pattern of the combination is intermittent.

For this dimension concept, we consider the patterning over a relatively constrained period as in task overlap. On one hand, it may be that there is just a single supported decision point in a significant workflow that incorporates many other (unsupported) decisions. If the form of the algorithmic contribution is not carefully aligned to the bulk of the workflow, then the cognitive burden of task-switching can undermine the effectiveness of the support. On the other hand, there may be a need for continuous algorithmic input over an extended period. In this situation, the algorithmic contribution is likely to be either visual or auditory to allow for continuous monitoring in the course of an extended task-set. The challenge lies in designing an interface that communicates sufficient salient information at any given moment while allowing for suitable resolution in the signal provided. We break down the *temporal patterning* dimension to distinguish four types as can be seen in Figure 7:

- *Sparse* - Instances where the human and algorithm combine on a decision are few, with significant time between them.
- *Occasional* - The use of (or the appearance of) algorithmic assistance is irregular and may be unanticipated.
- *Episodic* - Algorithmic contributions that occur at a reasonable frequency, but far short of continuous.
- *Continuous* - Continuous decision support describes a situation where the combination of human and algorithmic contributions is unbroken for an extended period.

As with the first two, each of these two new contextual dimensions has subtlety. They are challenging to define fully and distinctly because judging how much of a task is assisted is often evaluated by temporal means. But duration is, in reality, just one component of task burden. Cognitive load can play a more significant role. And parallel tasking trades off one for the other. Even more challenging is to define any points along these dimensions. There are no established scales of task overlap, while temporal patterning is a complex science that does not lend itself to a simple scale. For example, intermittency could involve a degree of unpredictability that is intrusive into patterns of normal work. Or it could imply regularity that fits well into the cognitive schedule of a human user. Nevertheless, we can grasp the essential concepts of each component, and acknowledge them as dimensions with the space. Even in the absence of fine-grained definitions, we argue that we should develop an early awareness of the existence and extent of the space that is being revealed.

#### Examples (Task overlap & temporal patterning) from situated studies

Among the situated studies, there are relatively few examples of systems supporting only sparse combination, and similarly with systems providing continuous support (Table 2). The bulk of systems are used to provide intermittent decision support. We often have to assume typical workflows in some cases, as even situated studies do not provide detailed ethnographic descriptions of the kind that would allow definitive classification. But there are some distinctive examples. From the middle of Table 2 (moderate task overlap with episodic patterning), Tschandl showed that a binary assist on the issue of malignancy in dermatology was less helpful since the clinician needed not only to decide this question, but also what diagnosis among seven possible types of lesion (Tschandl et al. 2020). The task overlap in one study treatment was too low, although it was high enough in others. Co-design and formative evaluation can help identify such issues early on in the development process.

From the upper left corner of Table 2 (very low overlap with sparse patterning), we have an example showing how context of use is important. Yang et al. (2019) discover that if multiple humans are together in discussion to make the decision, then the computer input can play an important empowering and nudging role - even without any formal delegation of control, without a large degree of task overlap and with only occasional use. To be effective in this scenario, it may well need to be ‘unremarkable’ as in this case. Otherwise, it risks adding to the cognitive burden of the human decision-makers and may be overall less effective.

At the other extreme, the work of Wang et al. (2018, 2019, 2020) demonstrates the intensive use of continuous algorithmic support in a situation where the task overlap is almost complete. The series of studies on endoscopic adenoma detection sits in a distinctive part of the overall contextual dimension space (very high overlap with continuous patterning). The design of the algorithmic input has to support extended effort on the part of the human operator. And the effect is to make the combination particularly effective at detecting micro polyps.

As can be seen from these examples, what constitutes a normal workflow for a given deployment context will affect whether temporal patterning is more or less intermittent. Retinopathy detection will be a regular occurrence in a specialist screening clinic, but the same task in a community general practice could be a sparsely occurring event.

**Table 2:** Dimensions for ‘Task overlap’ & ‘Temporal patterning’ and their frequency in the literature.

		Task overlap			
		$<<$50%	<50%	>50%	$>>$50%
Temporal patterning	Sparse	6	1		
	Occasional	3	5		2
	Episodic	1	11	11	21
	Continuous	3	1		4

### Informational proximity and informational overlap

*Informational proximity*, at first sight, is a simple indicator of relative physical location. But, in relation to decision-making, we should consider it an expression of the degree of friction associated with augmenting current information (Figure 8). One decision-maker may be physically or even geographically remote from a decision-context but yet have access to huge volumes of additional data. Another may be in the same room as a patient but have access to no records or results. The first, in our conception of this challenge space, could be considered more proximate to relevant information. While the person in front of the patient may be more remote from information that should influence a decision. *Informational proximity*, as we have defined it, highlights differences in the situated use of information in combined decision-making. We split *informational proximity* into two types, as shown in Figure 8 that nevertheless indicate a continuum:

- *Proximate* - Access to additional, good quality, digestible information relevant to the decision can be achieved with very low friction.
- *Remote* - Access to additional information relevant to the decision cannot be achieved without either significant effort, delay, quality issues or analytical obstacles.

*Informational overlap* is the degree to which two or more agents access a common portion of the total possible data relating to a decision (Figure 9). In many decision-contexts, the salience of different data elements is well-established. As a result, preparing for a decision may well involve assembling the same data ‘ingredients’ in most cases. But data dependence in medicine is discontinuous. So, what played a ‘peripheral’ role in one decision may turn out to become a critical variable, central to another case, even when facing what is nominally the same decision. Moreover, agents may have differential access over the total data. So which agent’s portion of data is critical in which case may or may not be predictable or consistent.

As a result of the non-overlapping areas of information, agents in a situation may well be expected to develop very distinct perspectives on a decision context. How and to what extent their informational fields diverge should therefore influence how their perspectives are combined. We divide *informational overlap* into three classes, as seen in Figure 9:

- *Disjoint* - The algorithm and the human possess completely different sets of information, meaning any decisions are made based on different perspectives and different sets of information.
- *Intersecting* - The algorithm and the human possess some combination of contiguous and disjoint information, allowing for some shared knowledge while also presenting information solely available to one part of the relationship.
- *Contiguous* - The algorithm and the human possess the same information, although not necessarily the same ability to utilise this information. An example would be both human and algorithm having the same view, but the algorithm having a much more highly tuned process of identifying problem areas.

By the distribution presented in Table 3, nearly three-quarters of situated studies involve agents with a degree of informational overlap (intersecting information sets). Although this figure hides a high variability in the degree of overlap within this intersecting classification, we note that the apparently less common cases (disjoint and contiguous information sets) might represent situations worthy of particular attention. We also see a large number (85%) of proximate systems - where the human agent may be readily able to augment core data, by access to additional sources, when necessary.

**Table 3:** Dimensions for ‘Informational proximity’ and ‘Information overlap’ and their frequency in the literature.

		Informational proximity	
		Proximate	Remote
Informational overlap	Disjoint	6	5
	Intersecting	36	5
	Contiguous	15	2

#### Examples (Informational proximity & informational overlap) from situated studies

In our first example, Bansal et al. (2018) evaluate not just an algorithm’s ability to detect sepsis, but their whole project’s ability to show benefit in clinically meaningful endpoints. The setting provides a clear example of how the approach to combination and the situated design of the machine-human interface is fundamental to translating from ‘bench to bedside’. The system follows a common pattern of supporting a proximate user with an output that is derived from a combination of information held at the bedside (clinical observations) and information from beyond the bedside (hence informationally intersecting).

A complement to the project reported by Bansal et al. is a 2020 detailed case study by Sendak et al. (2020a) which reports a system called Sepsis Watch implementing an intelligent alert component. The Sendak paper focuses on studying the real-world implementation of Sepsis Watch as a “socio-technical system requiring integration into existing social and professional contexts” (Sendak et al. 2020a, b). Key to the success of the project in addressing a situated decision problem was that the starting point was the recognised problem on which the core team sought improvement and from which a need or opportunity for algorithmic solution arose. In other words, this was not a bench design in search of its real-world application. In common with many alerting systems, the primary user (a dedicated nursing role actions and tracks each alerted case) is remote from the source of raw data, the unwell patient. And in common with many clinical systems, there is an intersection between the information available to the algorithm and the user.

The ophthalmology screening system described by Gulshan et al. (2019) is an example of a proximate contiguous informational space. The contiguous informational space results from the same images being used by the algorithm and the human grader. However, while this is a prospective study, its objective is to validate the performance of the algorithmic output. The study design has the AI system working in ‘shadow mode’ (see Section 2.3) and there is no in-workflow human recipient of the algorithmic output. So, this example shows there are challenges in detecting what counts as situated evaluation. The work itself validates a retinal fundus image classification algorithm in selecting cases of referable retinopathy from among diabetes patients. The prospective study design leads to some real-world challenges, but these are overcome and the results provide good quality evidence that the system generalises to a population of Indian patients.

A complement to the Gulshan et al. (2019) study is a (fully situated) follow-up study on the same system being deployed in Thailand (Beede et al. 2020). This in-depth study of the situational, technical, human and social factors surrounding real-world deployment raises challenges both for the design of the AI system and for the conduct of useful, ethical research. Issues not previously encountered included: significant variations in setting; technical issues with image quality; connection to cloud services; managing patient expectations if direct referral was indicated and changes required to existing workflows in order to incorporate the AI system. While few issues sit within the space of combination, all of them emphasise the need for situated evaluation of a proposed system.

### Input Influence and Output Representation Coverage

*Input influence* describes whether and how the actions of the human user influence the input to the algorithmic agent at inference time. Note that this is distinct from the input of training or reinforcement data which is intended to modify the model. Many system designs require user input to actively influence the algorithmic agent. For example, a video endoscopy system captures pictures inside the body as directed by the clinician. The user determines where the camera goes and how quickly it moves, so influencing the ingestion of data for the system’s decisions. It will be essential that a certain quality of image capture is attained for the decisions to be reliable, and the system design will need to take this into account. On the other hand, a system design may intentionally make it impossible for the algorithm to be influenced by the user - algorithm output may be driven purely by pre-determined data streams over which the user has no control. What may be less considered is the situation where the human user is able to influence the input to the algorithm without this being an intended feature of the system design.

We layout *input influence* as one of three types, visible in Figure 10:

- *Intentional influence* - The user intentionally influences the algorithmic input, in a way that is designed within the system.
- *Unintentional influence* - The user is able to influence the algorithmic input in a way that is not designed for.
- *No influence* - The user has no influence on the input data streams of the algorithmic agent. User influence is limited to what happens as a result of the output.

*Output representation* coverage indicates the ‘sidedness’ of the designed algorithm output. When engaging in combined decision-making together, humans rely on being able to give and get both an answer and its negation or complement (e.g., being able to say ‘yes’ and ‘no’). In considering the participation of an algorithmic agent, we need to know if it has full coverage in its output representation - or whether it will only ever give a limited coverage set of values (e.g., it can only say ‘yes’ or stay silent). A binary-valued decision outcome might be indicated by alternating the presence and absence of a positive algorithmic output. Or it might be indicated by a negative output being present or absent. Each of these makes no distinction between the system withholding a signal and it failing to operate. A more complete output representation coverage would include all possible logical values being expressible. We put *output representation* coverage for binary-valued decisions into three groups, as shown in Figure 11:

- *Positive* - The algorithm only outputs positive results, or only reports on positive hits within its decision space.
- *Negative* - The algorithm only outputs negative results, or only reports on negative hits within the decision space.
- *Both* - The algorithm reports both positive and negative hits within the decision space.

**Table 4:** Dimensions for Input influence and Output representation coverage and their frequency in the literature.

		Input influence		
		Intentional	Unintentional	No Influence
Output coverage	Positive	1	1	42
	Negative	1		1
	Both	5		18

It is clear from Table 4 that there is a large number of studies with a conventional design that does not allow user influence on input and produces only positive-directed signals. What is interesting about how the space is populated is that there is just one study in the unintentional input influence group, and there is a moderately sized group of studies in the intentional input influence group which nearly all have a full output representation coverage.

#### Examples (Input influence & output representation coverage) from situated studies

An important study that anticipates the greater use of Natural Language Processing in the form of speech recognition is that by Blomberg et al. (2021). Though this prospective trial did not demonstrate real benefit, it contributes to our understanding of the obstacles to be overcome. The authors describe how powerful the alert system is at detecting Out of Hospital Cardiac Arrest (OHCA) calls to an Emergency Medical Service. But translating better-than-human sensitivity into a support tool that helps improve human sensitivity requires consideration of many other factors that the human is required to attend to. This is also an example of a situation where there is *input influence* - the human user is creating part of the input for the system. An AI system listens in on the call in progress and can alert the human user to what might be an OHCA. But since the call is a two-way dialogue, the human user of the system is also feeding into the algorithm. This is not a design feature, so user input is not intended to affect what the system determines. But it is likely that there is an unintended effect. More research is needed on what can happen and what the implications are when users effectively have a two-way dialogue with a machine. This is another area which in which we see inevitable growth and development - but the interaction must be consciously developed and tested.

## Discussion

We introduced this paper with an outline of the *AI chasm* - a failure to translate from promising experiments to real-world benefit. This is a significant challenge for developers and designers of algorithmic systems, and the history of unmet expectations is not new. As part of the response to this situation, Cabitza and Zeitoun (2019) make a call to action on the need to demonstrate pragmatic and ecological validity in clinical AI. They outline a process that involves clinicians in evaluation against real-world end points. Animating the term *technovigilance* to parallel the forms of situated evaluation and reporting adopted in post-market pharmaceutical regulation, they make a case for earlier, situated and more continuous responsible assessment. In the culture of ‘real-world validation’, they advocate going beyond the initial technical question ‘can it work?’ to the pragmatic question ‘does it work?’ (beyond the bench) and the sustainability question ‘is it worth it?’ (Cabitza et al. 2019, p5).

Our framework complements this approach to validation. But it focuses on an earlier phase of the development process, by providing insight to both developers and evaluators on the difficulties of the first transition - going beyond the bench. Classifying spaces within the dimensions of human-machine combination is not just a theoretical exercise, but a necessary step towards practical application, to meet pressing practical and regulatory needs. Awareness of *how* humans will need, or be able, to combine with an intelligent system should inform system developers and should encourage formative and iterative situated evaluation throughout development as well as beyond deployment.

We have outlined the importance of attending to performance across all the processes that support combination - modelling, exchanging and resolving. While we acknowledge that past and current work has shown hugely impressive results in modelling, it is evident that those advances need to be matched by advances in exchanging and resolving if we are to make real-world progress. Specifically, bench studies that focus on machine performance are appropriate for models that operate in isolation, but they are not well suited to the development of models that specifically support exchanging and resolving. We have made the case that, in anticipation of situated evaluation, system developers should work with clinical end users as co-creators and attend to the contextual dimensions of the real combination space. They should seek to find ways of making appropriate attention possible (that is, default to unremarkable computing) so that algorithmic contributions are adjunct and timely; moreover, they should anticipate the need for dialogic exchange that supports convergence ahead of supporting resolution according to appropriate control relations.

Our examination of key writings on control, interaction, situatedness and decision-making have provided a theoretical focus for our review of studies. Our survey, in turn, shows that there are relatively few situated studies and fewer still that are prospective. Our reason for focusing attention on these, in spite of their low number, is because they provide evidence, not only of whether the systems under study provide real-world benefit, but also of how that benefit is to be leveraged from the combination of algorithmic systems with humans who are engaged in their daily work. We have drawn out, with examples, the ways in which our framework dimensions have been recognised implicitly by people working successfully to combine human and algorithmic contributions.

So, if developers and system designers achieved these successes without reference to a new framework, it could be argued that such a framework is unnecessary. But the relative dearth of translations beyond the bench, which is expressed in many commentaries and which is reflected in our survey, provides support for the contention that more conscious and consistent efforts need to be made to anticipate situated human-machine interaction with all the competing pressures of information relevance, timeliness, situational awareness, non-distraction, appropriate focus, task-switching, automation-bias and algorithmic aversion.

Approaching development and evaluation of algorithmic systems using the dimensions of combination we have outlined here can facilitate the assessment of, and improvement to, pragmatic and ecological validity that is needed for health systems, societies and patients. Making use of this perspective allows us to look at a proposed deployment and understand what we need to pay attention to in order to be confident it will work sufficiently well, and will continue to work sufficiently well in the future in a real-world setting, for it to be worthwhile. And once translation is accomplished, we can monitor it (vigilance) because we know the different dimensions along which it is operating.

Our typology essentially acts as a detailed map, guiding a conscious exploration of the functionalities and intended operational protocols of these systems. It allows for each model under our typology to be characterised by details of its ‘intended purpose’^1^ and situated constraints, opportunities and risks - going beyond the system’s immediate function of providing its answer to a decision question and encompassing the context and manner of its intended use.

Such awareness, shared with users, forms part of the essential framework that enables benefit without unseen costs. For example, it aligns with stipulations of the European AI Act, Art. 13 ‘Transparency and Provision of Information to Deployers’ (due to enter into force on 2 August 2026), which mandates comprehensive, clear, and comprehensible information for high-risk AI systems.

By specifying the ‘intended purpose’ and situated mode of operation (e.g., in model cards that specify the dimensions of combination), we ensure systems are used within their designed parameters, fortifying the bridge between theoretical insight and real-world application.

Our typology is designed as the outlines of a pragmatic tool, aimed at guiding designers, practitioners, and policymakers in the ethically sound and responsible development, deployment and use of AI in clinical settings. We do not believe it to be complete, but we argue that it provides a view on how such a nuanced classification on the dimensions of combination is essential for informed system design and evaluation.

## Limitations and future work

The task of laying a framework for understanding the dimensions of human-machine combination in situated clinical contexts is undoubtedly ambitious for a single study to tackle. Our analysis reveals a relative scarcity of situated studies that investigate in-depth the real-world application of human-AI combination in the clinical setting. This calls for more empirical research to understand how these combinations function in practice, beyond theoretical models. We look at studies across very different disciplines (clinical and computer science), and this presents challenges precisely because there is an underdevelopment of interdisciplinary work. So, one limitation is there not yet being a common evaluation standard. However, there is recent work that means this challenge is now being addressed (Natali et al. 2024).

Our derivation of dimensions has been driven by what we have found during our research. We have somewhat arbitrarily limited ourselves to eight dimensions as this appears to make best use of the information available, both in the background literature and the surveyed studies. There is no reason to suppose that these are the only dimensions - nor even that they will prove to be the most important. As such, we offer them as an indication of the space at this point in the evolution of human-machine combination work.

More specifically, our methodology lacks a robust method for parsing out distinct positions within a given dimension of human-machine interaction. This limitation is particularly evident in scenarios where control dynamics involve multiple humans and machines, challenging the common dyadic representation of a single human and single machine interacting. This also complicates the separation of dimensions, suggesting a need for more nuanced analysis to deepen and clarify our understanding of each one.

An obvious area for future work is to apply these dimensions during the development phase of an algorithmic model. Asking questions prompted by the framework about the different ways in which humans will work on their decision-making in the course of everyday work - and into which any new algorithm will need to fit - should inform development work itself. We intend to develop this application work in the coming period.

Two important and specific areas for future work are in order-effects for algorithmic suggestions (before or after a human decision) and in explanation-effects (whether they risk inducing over-reliance in a given context). These have been touched on only lightly in this paper (see Section 4.1). More work needs to be done on each. And it would be instructive to incorporate them into this framework.

A third area for future work is to explore any development that starts to occupy the peer space on the control spectrum. While there are no obvious signs of this at present, there is every reason to suppose it will be an area that is soon encroached upon - and that evaluation of theoretical designs will need to be carefully considered within a framework such as the one we present in this paper.

A fourth key area for future work involves expanding the dimensions of human-AI combination as they apply to the increasing adoption of LLMs in the clinical domain. Although we believe that LLM-based systems already fit into our dimensions - especially so for the *input-influence* and *informational overlap* dimensions (see Section 4.4) - nevertheless, a focused study on emerging, LLM-enabled combinations of humans and AI could enrich our framework by incorporating more explicit elements of dialogue, consensus-building, and interactive exploration.

In fact, the whole area for less structured interactions in human-AI combination remains under-explored. There are exceptions, such as Carrie Cai’s work presenting humans with the ability to adjust a range of concept representations. Ultimately, our analysis identifies a significant gap in the ‘peer space’ in Table 1. More research in this space could mimic human-to-human articulation work to a greater extent than rigid, structured protocols would allow. Such ‘unstructured’ integration, without anthropomorphising the machine, could afford to the human agents the flexibility to play to their natural strengths.

## Conclusion

In this work, we have shown that the *AI chasm* described in the introduction is evidenced in the literature. We propose that crossing the *AI chasm* requires exploration that maps out the terrain - where there are gaps in our design and evaluation work. We argue that the insight revealed through our *contextual dimensions of combination* gives us a sense of the ground that needs to be covered.

These *dimensions of combination* are not all new conceptually. But for the first time they are brought together to provide a framework within which the research community can approach this challenge. Previous work has looked at the spectrum of control but without specifying it in the way we have done here. And without putting it into a wider context of how combination is effected. With this novel framework, we can expand our insight on how combined human-machine decision-making takes place when systems are actually deployed. And it then becomes possible to tease out how situated particulars significantly affect any meaningful measure of validity. We believe our contention that system designs will increasingly occupy the empty ‘peer’ areas revealed in Table 1 is uncontroversial, but how we design and evaluate for these different terrains is key since each dimension makes a contribution.

The emergent combination space itself is multi-dimensional and highly variable, but must be considered as the context within which combined human-AI decision-making currently exists and the terrain over which it will expand.

This space of combination is where any dialogue, exchange, convergence and resolution will take place. We must be aware of, and research carefully, the informational and interactional space humans and machines share.

## Data Availability

No datasets were generated or analysed during the current study.

## Footnotes

1 “a description of what the device’s functionality is intended to achieve. Typically, for software products this should be described as a process with the product inputs and outputs specified, including the purpose and actions to be taken by the user.”

## Declarations

### Open Access

For the purpose of Open Access, the author has applied a CC BY licence to any Author Accepted Manuscript (AAM) version arising from this submission.
